# Coronary Artery Bypass Surgery in Brazil: Analysis of the National Reality Through the BYPASS Registry

**DOI:** 10.21470/1678-9741-2018-0313

**Published:** 2019

**Authors:** Rodrigo Pereira Paez, Nelson Américo Hossne Junior, José Amalth do Espírito Santo, Otavio Berwanger, Renato Hideo Nakagawa Santos, Renato Abdala Karam Kalil, Fabio B. Jatene, Alexandre Biasi Cavalcanti, Alexandre Cabral Zilli, Luiz Carlos Bettiati Jr, Fernando Augusto Marinho dos Santos Figueira, Stephanie Steremberg Pires D'Azevedo, Marcelo José Ferreira Soares, Marcio Pimentel Fernandes, Roberto Vito Ardito, Renata Andrea Barberio Bogdan, Valquíria Pelisser Campagnucci, Diana Nakasako, Clarissa Garcia Rodrigues, Anilton Bezerra Rodrigues Junior, Marcelo Matos Cascudo, Fernando Antibas Atik, Elson Borges Lima, Vinicius José da Silva Nina, Renato Albuquerque Heluy, Lisandro Gonçalves Azeredo, Odilon Silva Henrique Junior, José Teles de Mendonça, Katharina Kelly de Oliveira Gama Silva, Marcelo Pandolfo, José Dantas de Lima Júnior, Renato Max Faria, Jonas Gonçalves dos Santos, Guilherme Henrique Biachi Coelho, Sergio Nunes Pereira, Roberta Senger, Enio Buffolo, Guido Marco Caputi, Juliana Aparecida Borges de Oliveira, Walter J. Gomes, BYPASS Registry Study Group

**Affiliations:** 1 Hospital São Paulo, Escola Paulista de Medicina da Universidade Federal de São Paulo, (EPM - UNIFESP), São Paulo, SP, Brazil.; 2 Instituto de Pesquisa do Hospital do Coração (IP - HCor), São Paulo, SP, Brazil.; 3 Instituto de Cardiologia do Rio Grande do Sul - Fundação Universitária de Cardiologia, Porto Alegre, RS, Brazil.; 4 Cardiovascular Surgery Division, Instituto do Coração do Hospital das Clínicas da Faculdade de Medicina da Universidade de São Paulo (InCor-HCFMUSP), São Paulo, SP, Brazil.; 5 Hospital de Caridade São Vicente de Paulo, Jundiaí, SP, Brazil.; 6 Instituto de Medicina Integral Professor Fernando Figueira (IMIP), Recife, PE, Brazil.; 7 Hospital de Base - FUNFARME e FAMERP, São José do Rio Preto, SP, Brazil.; 8 Instituto de Moléstias Cardiovasculares (IMC), São José do Rio Preto, SP, Brazil.; 9 Irmandade da Santa Casa de São Paulo (INCT-HPV/Faculdade de Ciências Médicas da Santa Casa de São Paulo), São Paulo, SP, Brazil; 10 Instituto do Coração de Natal, Natal, RN, Brazil.; 11 Instituto de Cardiologia do Distrito Federal, Brasília, DF, Brazil.; 12 Hospital Universitário da Universidade Federal do Maranhão (HU/UFMA), São Luís, MA, Brazil.; 13 Hospital Evangélico, Cachoeiro de Itapemirim, ES, Brazil.; 14 Hospital do Coração de Sergipe, Aracaju, SE, Brazil.; 15 Instituto de Cirurgia Cardiovascular (ICCV)/Hospital Nossa Senhora da Salete, Cascavel, PR, Brazil.; 16 Hospital Wilson Rosado, Mossoró, RN, Brazil.; 17 Hospital Bosque da Saúde, São Paulo, SP, Brazil.; 18 Hospital Universitário de Santa Maria, Santa Maria, RS, Brazil.; 19 Hospital do Coração (HCor), São Paulo, SP, Brazil.

**Keywords:** Registries, Database, Cardiovascular Surgical Procedures, Cardiac Surgical Procedures, Coronary Artery Bypass Grafts, Myocardial Revascularization

## Abstract

**Introduction:**

Coronary artery bypass grafting (CABG) is the most frequently performed heart surgery in Brazil. Recent international guidelines recommend that national societies establish a database on the practice and results of CABG. In anticipation of the recommendation, the BYPASS Registry was introduced in 2015.

**Objective:**

To analyze the profile, risk factors and outcomes of patients undergoing CABG in Brazil, as well as to examine the predominant surgical strategy, based on the data included in the BYPASS Registry.

**Methods:**

A cross-sectional study of 2292 patients undergoing CABG surgery and cataloged in the BYPASS Registry up to November 2018. Demographic data, clinical presentation, operative variables, and postoperative hospital outcomes were analyzed.

**Results:**

Patients referred to CABG in Brazil are predominantly male (71%), with prior myocardial infarction in 41.1% of cases, diabetes in 42.5%, and ejection fraction lower than 40% in 9.7%. The Heart Team indicated surgery in 32.9% of the cases. Most of the patients underwent cardiopulmonary bypass (87%), and cardioplegia was the strategy of myocardial protection chosen in 95.2% of the cases. The left internal thoracic artery was used as a graft in 91% of the cases; the right internal thoracic artery, in 5.6%; and the radial artery in 1.1%. The saphenous vein graft was used in 84.1% of the patients, being the only graft employed in 7.7% of the patients. The median number of coronary vessels treated was 3. Operative mortality was 2.8%, and the incidence of cerebrovascular accident was 1.2%.

**Conclusion:**

CABG data in Brazil provided by the BYPASS Registry analysis are representative of our national reality and practice. This database constitutes an important reference for indications and comparisons of therapeutic procedures, as well as to propose subsequent models to improve patient safety and the quality of surgical practice in the country.

**Table:** 

Abbreviations, acronyms & symbols				
ADA	= Anterior descending artery		GCP	= Good Clinical Practices
ARDS	= Adult respiratory distress syndrome		ITA	= Internal thoracic artery
BYPASS	= Brazilian Registry of Adult Patients UndergoingCardiovascular Surgery		LAD	= Left anterior descending
CABG	= Coronary artery bypass grafting		LITA	= Left internal thoracic artery
CAD	= Coronary artery disease		LVEF	= Left ventricular ejection fraction
COPD	= Chronic obstructive pulmonary disease		MI	= Myocardial infarction
CPB	= Cardiopulmonary bypass		NYHA	= New York Heart Association classification for heart failure
EACTS	= European Association for Cardio-Thoracic Surgery		PCI	= Percutaneous coronary intervention
EAPC	= European Association of Percutaneous Cardiovascular Interventions		RITA	= Right internal thoracic artery
ESC	= European Society of Cardiology		SBCCV	= Sociedade Brasileira de Cirurgia Cardiovascular
FREEDOM	= Future Revascularization Evaluation in Patients withDiabetes Mellitus: Optimal Management of Multivessel Disease		STS	= Society of Thoracic Surgeons
			SVG	= Saphenous vein grafts
			SYNTAX	= Synergy Between Percutaneous Coronary Intervention with TAXUS and Cardiac Surgery

## INTRODUCTION

The Brazilian Registry of Adult Patients Undergoing Cardiovascular Surgery - the BYPASS Project - is an ongoing database established in 2015 by the Sociedade Brasileira de Cirurgia Cardiovascular (SBCCV) and aimed to collect perioperative and postoperative data from patients undergoing cardiac surgery in Brazil.

The first report of the BYPASS revealed that coronary artery bypass grafting (CABG) is the most frequently performed cardiac surgery in Brazil, encompassing 54.1% of the cases^[1]^. CABG remains the standard of care for management of patients with coronary artery disease, particularly in high-risk patients with multivessel disease, diabetics, aged 65 years or more, with left main stem or with impaired left ventricular function.

Previous analyses of results of CABG patients in Brazil were derived from single centers, a group of hospitals or defective databases, failing to reach national representativeness. Recent international guidelines recommend that national societies establish their own database on the practice and results of CABG and, in anticipation of this, the BYPASS registry was introduced in 2015^[2,3]^.

To provide a comprehensive understanding of the scenario of cardiovascular surgery in Brazil, this analysis presents the clinical profile and outcomes of patients undergoing CABG and enrolled in the BYPASS project, assessing the data harvested from the initial cohort of patients included.

## METHODS

This study was approved by the Research Ethics Committee of the Universidade Federal de São Paulo (UNIFESP - Registration number 1143/2017) and compiled data from the Bypass Registry, an observational and prospective study, with longitudinal follow-up. This report comprises a cross-sectional study, evaluating patients undergoing first-time isolated CABG.

Population and Locations

The participation of cardiovascular centers in this project was voluntary and involved 17 institutions throughout the country and located in the following regions: Southeast^[4]^, Northeast^[5]^, South^[3]^, and Central-West^[1]^.

The project is partnered with the HCor Research Institute, responsible for collecting, analyzing and auditing the data.

A total of 2292 patients were analyzed, included until November 2018. The population consisted of patients older than 18 years of age, submitted to first-time isolated CABG, without other associated procedures. The informed consent form was signed by every patient included in the study and the data collection followed the National Clinical Research regulation standards as well as the Document of the Americas and Good Clinical Practice (GCP). The Bypass Registry was approved by the Ethics and Research Committee of the coordinating center and each participating institution.

### Profile and Outcomes

The main variables assessed were demographic data, clinical presentation, risk factors, medications in use, left ventricular ejection fraction (LVEF), evaluated by echocardiography, and laboratory tests. Intraoperative data recorded operative time, use of cardiopulmonary bypass (CPB) and cardioplegia, type of grafts harvested, and preferred anastomotic techniques (direct, sequential or Y-composite grafts). Postoperative data included incidence of the following complications: stroke, major bleeding requiring reoperation, utilization of blood products, vasoplegia, arrhythmia, myocardial infarction, low cardiac output, use of vasoactive drugs, mechanical ventilation longer than 24 hours, kidney failure, need for transfusion, infection, and hospital mortality.

### Statistical Analysis

The quantitative variables were described by mean ± standard deviation, and the qualitative variables were presented in form of absolute and relative frequency. Statistical analyses were performed with the Statistical Package R version 3.3.2 (R Foundation for Statistical Computing).

## RESULTS

Epidemiological data and relative risk factors for heart disease are presented in Table 1. Patients referred to CABG in Brazil were predominantly male (71%), mean age 63.5±9.6 years, with recent myocardial infarction (MI) in 41.1% of the cases.

**Table 1:** Demographics data.

	Total (n=2292)
Sex (female)	665/2292 (29%)
Age; mean±SD (years)	63.5±9.6
Family history	867/2292 (37.8%)
Diabetes	973/2292 (42.5%)
Dyslipidemia	1250/2292 (54.5%)
Hypertension	1937/2292 (84.5%)
Infarction	941/2292 (41.1%)
Previous PCI	289/2292 (12.6%)
Previous heart surgery	48/2292 (2.1%)
CVA	93/2292 (4.1%)
PAD	170/2292 (7.4%)
Heart failure	328/2292 (14.3%)
NYHA (1)	42/312 (13.5%)
NYHA (2)	181/312 (58%)
NYHA (3)	82/312 (26.3%)
NYHA (4)	7/312 (2.2%)
Kidney failure	111/2292 (4.8%)
Dialysis	18/109 (16.5%)
Cardiac arrest	45/2292 (2%)
Current smoker	309/2292 (13.5%)
Ex-smoker	570/2286 (24.9%)
Arrhythmia	117/2292 (5.1%)
Pacemaker	20/2292 (0.9%)
COPD	128/2292 (5.6%)
EF <40%	185/1904 (9.7%)

COPD=chronic obstructive pulmonary disease; CVA=cerebrovascular accident; EF=ejection fraction; NYHA=New York Heart Association classification for heart failure; PAD=peripheral artery disease; PCI=percutaneous coronary intervention

Table 2 depicts the reimbursement source, whether public or private, and the setting in which the patients were operated on; *i.e*., elective or urgent surgery. Most of the patients were admitted through the public healthcare system (84.4%) and operated under elective conditions (77.7%).

**Table 2:** Reimbursement source and preoperative conditions.

	Total (n=2292)
Reimbursement source	
Public healthcare system	1934/2292 (84.4%)
Private - Health insurance	358/2292 (15.7%)
Type of procedure	
Elective surgery	1781/2292 (77.7%)
Urgent surgery	478/2292 (20.9%)
Emergency surgery	32/2292 (1.4%)
Surgery indicated by the Heart Team	754/2292 (32.9%)
Clinical conditions	
Stable	2212/2292 (96.5%)
Unstable	73/2292 (3.2%)
Critical	7/2292 (0.3%)

Intraoperative variables regarding surgical access, use of cardiopulmonary bypass, myocardial protection, use of internal thoracic artery and other arterial grafts, Y- or sequential grafts were collected according to Table 3.

**Table 3:** Intraoperative characteristics.

	Total (n=2292)
Open-heart surgery	2291/2292 (100%)
Minimally invasive surgery	1/2292 (0%)
Use of cardiopulmonary bypass	1994/2292 (87%)
Cardioplegia	1899/1994 (95.2%)
Grafts	
SVG	1925/2289 (84.1%)
No SVG (only arterial grafts)	364/2289 (15.9%)
Only SVG	176/2289 (7.7%)
Use of LITA	2083/2289 (91%)
Use of RITA	129/2289 (5.6%)
One ITA	
Use of LITA or RITA	1994/2289 (87.1%)
Use of Only LITA or RITA	300/2289 (13.1%)
Two ITA	
Use of LITA and RITA	109/2289 (4.8%)
Use of Only LITA and RITA	48/2289 (2.1%)
Use of radial artery	25/2289 (1.1%)
Treated vessels; median [quartiles]	3 [2 - 4] (n=2292)

ITA=internal thoracic artery; LITA=left internal thoracic artery; RITA=right internal thoracic artery; SVG=saphenous vein graft

Intraoperative clinical events, such as bleeding, vasoplegia, arrhythmias, infarction [defined as an elevation of more than 10 times the upper limit of troponin T], use of vasopressors and intraoperative death are shown in Table 4. Postoperative follow-up was also analyzed in view of the main indicators and complications that may occur, according to Table 5.

**Table 4:** Intraoperative complications.

	Total (n=2292)
Myocardial infarction	11/2292 (0.5%)
Major bleeding	119/2292 (5.2%)
Transfusion	539/2292 (23.5%)
Postperfusion syndrome	15/2292 (0.7%)
Arrhythmia	70/2292 (3.1%)
Low cardiac output	102/2292 (4.5%)
Use of vasoconstrictors	1137/2292 (49.6%)
Intraoperative death	12/2292 (0.5%)

**Table 5:** Postoperative intra-hospital data.

	Total (n=2292)
Reoperation	52/2280 (2.3%)
Major bleeding	62/2280 (2.7%)
PCI	5/2280 (0.2%)
Mechanical ventilation more than 24h	120/2280 (5.3%)
Tracheostomy	12/2280 (0.5%)
ARDS	33/2280 (1.4%)
Low cardiac output	73/2280 (3.2%)
Kidney failure	84/2280 (3.7%)
Dialysis	30/84 (35.7%)
Coagulopathy	23/2280 (1%)
Transfusion	446/2280 (19.6%)
Arrythmias	336/2280 (14.7%)
Need for pacemaker	91/2280 (4%)
Infection	118/2280 (5.2%)
Myocardial infarction	27/2280 (1.2%)
Vasoplegic syndrome	27/2280 (1.2%)
Death	64/2292 (2.8%)
Heart failure	29/2280 (1.3%)
CVA	28/2280 (1.2%)

ARDS=adult respiratory distress syndrome; CVA=cardiovascular accident; PCI=percutaneous coronary intervention

Most of the patients underwent CPB (87%), and cardioplegia was the strategy of myocardial protection chosen in 95.2% of the cases.

The left internal thoracic artery (LITA) was used in 91% of the cases; the right internal thoracic artery (RITA), in 5.6%; and radial artery in only 1.1%. Total arterial revascularization was performed in 15.9%, and a significant portion (13.1%, 300 of 2289 patients) received an isolated internal thoracic artery graft to the left anterior descending (LAD) artery.

Saphenous vein grafts (SVG) were used in up to 84.1% of patients. There were 7.7% of patients revascularized with vein grafts only, without arterial grafts. The median number of vessels treated was 3^[2,3]^.

The in-hospital mortality rate was 2.8%, and the incidence of clinically relevant cerebrovascular accident was 1.2%.

## DISCUSSION

Coronary artery disease is one of the leading causes of mortality and morbidity in the 21^st^ century^[4]^. In Brazil, approximately 30% of people die from cardiovascular causes according to data from the Unified Health System (DATASUS)^[5]^.

In 2014, CABG celebrated six decades since Kolesov's direct coronary approach for myocardial revascularization. And the technique continues to evolve at all times, from the onset of the worldwide experience without CPB, to the advent of CPB, returning to new scenarios without the use of CPB^[6]^; from the use of the saphenous vein to the use of the internal thoracic artery, including sequential anastomoses, until coronary artery bypass grafting completely performed with arterial grafts^[7]^, with graft quality control by Doppler graft flow measurement^[4]^.

In patients with stable chronic disease, CABG is indicated to relieve symptoms and/or improve prognosis^[8]^. CABG surgery has well established indications and benefits when it comes to left main artery disease, multivessel coronary artery disease, diabetes, proximal LAD occlusion and left ventricular dysfunction^[9-12]^.

In recent decades, hospitals and surgeons have been called upon to present their findings to the medical community and society. The European EuroSCORE study showed general mortality in cardiac surgery around 3.1%, while the American multicenter Society of Thoracic Surgeons (STS), around 2.0%^[13,14]^. Hannan et al.^[15]^, analyzing 49,830 patients undergoing CABG surgery in New York state between 2001 and 2004, found mortality around 1.19%, stroke around 1.2%, transmural infarction 0.5% sternal infection 0.9%, reoperation for bleeding 1.9%, sepsis 1.0%, renal dialysis 1.4%, respiratory failure 3.7%, and reoperation 1.2%.

In our country, Silva et al.^[16]^ showed an operative mortality rate of 4.0% in patients operated by the Unified Health System (SUS) at the Hospital Beneficência Portuguesa in São Paulo, while Fortes et al.^[16,17]^ showed an average mortality of 7.4% in patients referred for CABG in a university hospital in northeastern Brazil. TotalCor Hospital, a private hospital focused on cardiology, points to a mortality rate of 1.7%. The profile of the patient operated in this private hospital is also favorable, with an expected mortality rate of 0.7%^[18]^.

The BYPASS Registry is the first study of a Brazilian multicenter database with national representativity. The records strengthen that Brazilian patients, in general, have little access to primary prevention and surgery was indicated after heart attacks, heart failure and ventricular dysfunction. Brazilian patients have unsatisfactory outpatient follow-up and are generally poorly medicated, either due to poor adherence or lack of medication in the public healthcare system.

Since it is a voluntary participation, currently 17 Brazilian centers spontaneously provide their data to the BYPASS Registry. It should be remembered that the STS database also started with few centers and still does not reach all centers yet, but around 90%. Over the years, the participation of centers increased due to the benefits of having their results analyzed and receiving advice to improve hospital processes focused on surgical quality and patient safety. Currently, the STS participating center is a recognized center for quality control and continuous improvement. The TotalCor Hospital in São Paulo provides data for the STS since 2011 and has since reached a decrease in the time of mechanical ventilation and hospital stay through the guidelines received^[18]^. Since its first publication in 2018, the BYPASS study has had great adherence of cardiac surgeons, increasing from 945 to 2292 patients undergoing CABG in follow-up. The BYPASS study also seeks to be an evaluator and consultant in the process of improvement in cardiovascular surgery in Brazil.

Diabetes was more prevalent in patients undergoing revascularization in Brazil, when compared to other international registries, such as the SYNTAX (Synergy Between Percutaneous Coronary Intervention With TAXUS and Cardiac Surgery) trial^[19]^. FREEDOM (Future Revascularization Evaluation in Patients with Diabetes Mellitus: Optimal Management of Multivessel Disease) trial defined bypass surgery as the standard treatment for diabetic and multivessel patients, mainly directing these patients for surgery.

Less than one-third of the reported patients undergoing revascularization surgery were female. Approximately 12% of the patients who underwent surgery had previously undergone percutaneous coronary interventions, an issue that deserves further investigation regarding the quality of the revascularization provided to these patients.

The vast majority of patients (84.4%) were operated in the public healthcare system, which draws attention since approximately 30% of Brazilians have private health insurance^[20]^. Such information suggests that many patients with access to private health care are operated on public hospitals, or that patients who have access to private health care initially avoid being referred to surgery.

The analysis showed that operative mortality in CABG in Brazil was slightly higher compared to North American and European centers. This finding is remarkable, given the limited budget, resources and infrastructure of the health system, both private and public, as well as operating rooms and intensive care units, both in basic equipment and human resources.

Brazilian surgeons currently prefer to perform on-pump CABG, the technique employed in 87% of the cases. In this scenario, cardioplegia has been the preferred strategy for myocardial protection (95.2%). This is an amazing finding in a country that pioneered the development of off-pump coronary surgery. Minimally invasive or robotic revascularization is not routinely performed in Brazil.

Major complications such as death, cerebrovascular accident, and myocardial infarction were similar to those of international studies. Major bleeding requiring reoperation, arrhythmias, kidney failure, infection and heart failure were also similar.

Vasoplegia was uncommon (0.7%) in this study. It is a well-known complication in the postoperative period of cardiovascular surgery, defined by hemodynamic collapse similar to septic shock, characterized by decreased systemic vascular resistance, increased cardiac index and severe hypotension, despite the use of vasoactive drugs, occurring in the first postoperative hours^[21]^.

The use of sequential anastomoses is a strategy to minimize aortic manipulation, but also to reduce CPB time and aortic cross-clamping.

Bilateral internal thoracic arteries (ITA) were used only in 4.8% of the patients, a practice more frequent than that reported by the STS database (4.1%), but fair below the European practice (10%)^[22,23]^. Only 16.9% of the patients had total arterial graft, of which 69% of the patients received only one ITA graft.

The radial artery was seldom used (1.1%); and 7.7% of the patients received only the saphenous vein as a graft. The saphenous vein graft was widely used (84.1%), similar to Europe (84.4%) and the United States (95.1%)^22-24^. The time spent for preparation of the grafts can influence surgical teams with high volume to prefer the saphenous vein use instead of harvesting the second internal thoracic artery. Injury of the LITA during the preparation may also induce the surgeon to perform saphenous surgery instead of preparing the RITA.

Only 32.9% of the patients had their procedures indicated by the Heart Team, being a growing trend and demands of medical guidelines that patients have indication for cardiac procedures discussed and based on scientific evidence. Since the first report, the BYPASS study has seen a large increase in the participation of the Heart Team in the indication of cardiac surgeries^[1]^.

Understanding the differences between the surgical strategy of the Brazilian surgeon and that of other countries, even with regional differences, may guide and suggest improvements in the routines adopted by different centers^[25,26]^.

The experience of international databases shows that its continuous data management brings long-term benefits, optimizing hospital practices and directing preventive policies. The dissemination of the BYPASS Registry and the invitation to surgeons to provide their data have contributed to the growth of the database. The adherence of several Brazilian centers to provide their data to the BYPASS Registry should be encouraged as part of a process of continuous medical improvement and patient safety. Policies should be designed to help fund national registries and stimulate surgeons to provide their data.

## CONCLUSION

The CABG data in Brazil provided by the BYPASS Registry analysis are, so far, the most representative of our national reality and practice. This database constitutes an important reference for indications and comparisons of therapeutic procedures, as well as to propose subsequent models to improve patient safety and the quality of surgical practice in the country.

**Table:** 

Authors' roles & responsibilities	
WJG, JAES, JABO, OB, ABC, FBJ	Conception and study design; data management; manuscript redaction or critical review of its content; final manuscript approval
Other authors	Substantial contributions to the conception or design of the work; or the acquisition, analysis, or interpretation of data for the work; final manuscript approval
